# Assessment of myocardial injury after reperfused infarction by T1ρ cardiovascular magnetic resonance

**DOI:** 10.1186/s12968-017-0332-z

**Published:** 2017-02-15

**Authors:** Rutger H. Stoffers, Marie Madden, Mohammed Shahid, Francisco Contijoch, Joseph Solomon, James J. Pilla, Joseph H. Gorman, Robert C. Gorman, Walter R.T. Witschey

**Affiliations:** 1 Department of Radiology, Perelman School of Medicine, University of Pennsylvania, 1 Silverstein 3400 Spruce Street, Philadelphia, PA USA 19104; 2 Gorman Cardiovascular Research Group, University of Pennsylvania, Philadelphia, PA USA

## Abstract

**Background:**

The evolution of T1ρ and of other endogenous contrast methods (T2, T1) in the first month after reperfused myocardial infarction (MI) is uncertain. We conducted a study of reperfused MI in pigs to serially monitor T1ρ, T2 and T1 relaxation, scar size and transmurality at 1 and 4 weeks post-MI.

**Methods:**

Ten Yorkshire swine underwent 90 min of occlusion of the circumflex artery and reperfusion. T1ρ, T2 and native T1 maps and late gadolinium enhanced (LGE) cardiovascular magnetic resonance (CMR) data were collected at 1 week (*n* = 10) and 4 weeks (*n* = 5). Semi-automatic FWHM (full width half maximum) thresholding was used to assess scar size and transmurality and compared to histology. Relaxation times and contrast-to-noise ratio were compared in healthy and remote myocardium at 1 and 4 weeks. Linear regression and Bland-Altman was performed to compare infarct size and transmurality.

**Results:**

Relaxation time differences between infarcted and remote myocardial tissue were ∆T1 (infarct-remote) = 421.3 ± 108.8 (1 week) and 480.0 ± 33.2 ms (4 week), ∆T1ρ = 68.1 ± 11.6 and 74.3 ± 14.2, and ∆T2 = 51.0 ± 10.1 and 59.2 ± 11.4 ms. Contrast-to-noise ratio was CNR_T1_ = 7.0 ± 3.5 (1 week) and 6.9 ± 2.4 (4 week), CNR_T1ρ_ = 12.0 ± 6.2 and 12.3 ± 3.2, and CNR_T2_ = 8.0 ± 3.6 and 10.3 ± 5.8. Infarct size was not significantly different for T1ρ, T1 and T2 compared to LGE (*p* = 0.14) and significantly decreased from 1 to 4 weeks (*p* < 0.01). Individual infarct size changes were ∆T1ρ = −3.8%, ∆T1 = −3.5% and ∆LGE = −2.8% from 1 – 4 weeks, but there was no observed change in infarct size for T2 or histologically.

**Conclusions:**

T1ρ was highly correlated with alterations left ventricle (LV) pathology at 1 and 4 weeks post-MI and therefore it may be a useful method endogenous contrast imaging of infarction.

**Electronic supplementary material:**

The online version of this article (doi:10.1186/s12968-017-0332-z) contains supplementary material, which is available to authorized users.

## Background

Ischemic heart disease is an enormous health and economic burden and the most common cause of death throughout the world [1]. A devastating manifestation is acute myocardial infarction (MI) which results in myocardial loss and precipitates a cascade of events including myocardial scarring, adverse left ventricular (LV) remodeling, heart failure and death. While late gadolinium enhanced (LGE) cardiovascular magnetic resonance (CMR) can detect myocardial fibrosis, there is significant interest in non-gadolinium contrast or endogenous contrast methods to spatially map infarcted tissue, detect recent ischemic injury and edema, or assess injury in patients with insufficient renal function who cannot receive contrast agents [2–5].

T1ρ (“T-one-rho”) CMR has recently emerged as an endogenous contrast method for quantitative imaging of myocardial injury [6–13]. T1ρ is called the longitudinal relaxation time in the rotating frame and it uses continuous low amplitude radiofrequency pulses to suppress low frequency background contributions to relaxation that obscure image contrast between infarct and normal myocardial tissue. Unlike conventional relaxation times (T1 and T2), the nuclear magnetization is locked along the radiofrequency field and does not undergo normal T2 or T1 relaxation. In comparison to T2, T1ρ was reported in ex vivo studies to have superior dynamic range between infarcted and remote myocardium, permitting better detectability of fibrosis. However, there is limited information about the evolution of T1ρ after reperfused MI and comparison with other endogenous contrast methods T1 and T2 and LGE.

We conducted a serial study of reperfused infarction in pigs to monitor T1ρ at 1 and 4 weeks post-MI. The objectives were to compare T1ρ relaxation time changes in ischemic tissue with native T1, T2 and LGE and associate each with infarct size and transmurality. Finally, imaging results were correlated with fibrosis using histological data.

## Methods

### Animal care

Yorkshire swine (*n* = 10) were procured for this study. During all procedures, sedation was induced with intramuscular ketamine, endotracheal intubation was performed, and the animal was maintained with a mixture of isoflurane 1-2% and oxygen with a ventilator tidal volume of 20 mL/kg (Drager anesthesia monitor, North American, Dragor, Telford, PA). Anesthesia and animal temperature was closely monitored for the duration of surgical and imaging procedures to maintain a constant physiologic state. Arterial access was obtained at the carotid artery for measurement of intraventricular pressure (Millar Instruments, Houston TX). Venous access was obtained at the internal jugular veins for administration of medication. After each procedure, the animal was weaned from anesthesia and transported to the recovery room. Upon completion of the terminal CMR study, the animal was returned to the operating room for euthanasia and tissue harvest.

### Experimental protocol

**Fig. 1:**
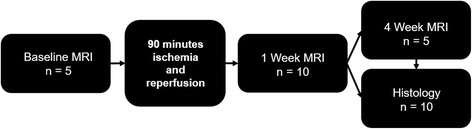
Experimental design. *N* = 10 animals underwent ischemia reperfusion and all animals had a 1 week CMR. Histology was performed in five animals immediately after 1 week CMR and 4 week CMR was performed in the other five animals

Ten pigs underwent 90 min of coronary artery occlusion and were randomized into two groups: group I, a 1-week post-MI terminal study (*n* = 5) and II, a 4-week terminal study (*n* = 5). Furthermore, five animals (group II; *n* = 5) underwent a baseline CMR immediately prior to coronary artery occlusion. The experimental model was chosen to emulate human post-percutaneous coronary intervention ischemia injury. All the animals of the 4 week group underwent both 1 and 4 week CMR. Figure 1 depicts an overview of the experimental protocol.

A prophylactic antiarrhythmic regimen of 150 mg amiodarone, 1 mg/kg lidocaine and 1 g magnesium sulfate was administered intravenously. A left thoracotomy was performed and the pericardium was opened. One or more coronary snares were positioned at the branches of the circumflex artery to induce an infarction of approximately 20% LV size. The dimensions of the infarction were determined by visible color changes at the epicardium. The exact position of the ligation sutures were decided upon after gross inspection of arterial anatomy, unique to the animal. Ischemia was confirmed by visible color changes in the ischemic region and ST segment elevations on ECG. After 90 min of ischemia, the coronary snare was removed, an inter-costal nerve block was performed with bupivacaine at the surgical site, and the chest was closed in muscle layers.

### Cardiovascular magnetic resonance protocol

CMR studies were performed on a 1.5 T whole-body system (Avanto; Siemens Healthcare, Erlangen, Germany) with 40 mT/m gradient and 12 channel RF receiver arrays. Intraventricular pressure was interfaced to physiological monitoring software and filtered to facilitate dual respiratory and cardiac gating (LabView, National Instruments, Inc., Austin Texas). All 2D images were acquired in the short-axis during breath-holding and 3D with dual cardiac and respiratory gating. Breath-holding was performed by temporarily disabling the animal ventilator.

2D T1ρ single-shot balanced steady-state free precession (bSSFP) sequences were performed using a spin echo, spin lock (SL) T1ρ pulse cluster (90_x_ – SL_y_ - 180_y_ – SL_-y_ - 90_-x_) [8, 14, 15]. T1ρ images were acquired with different TSL times using the following parameters: TSL = 2, 10, 18, 26, 34, 42, 50 ms, B_1_ = 500 Hz, spatial resolution = 1.4 × 1.4 mm^2^, slice thickness = 6 mm, flip angle = 70°, TE = 1.45 ms, TR = 2.9 ms, N_Seg_ = 55, bandwidth = 900 Hz/pixel, linear k-space phase encoding ordering, parallel imaging with acceleration factor = 2, 34 reference k-space lines obtained in a separate heartbeat, and four heartbeats for T1 relaxation between shots. The T1ρ amplitude was set at the highest available within scanner specific absorption rate limits (B_1_ = 500 Hz).

2D T2 maps were obtained using a single-shot T2 prepared (90_x_ – 180_y_ – 90_-x_) bSSFP sequence and 8 images were acquired with different contrast times TE = 2, 10, 18, 26, 34, 42, 50 ms using the same readout as with the T1ρ images.

2D T1 maps were obtained with a modified Look-Locker sequence, utilizing a single-shot acquisition with eight inversion times (two inversion pulses: five images obtained after inversion 1, 10 T1 recovery beats, and three images obtained after inversion two) [16]. Other parameters were: spatial resolution = 1.4 × 1.4 mm^2^, slice thickness = 6 mm, flip angle = 35°, TE = 1.2, TR = 2.4 ms, N_Seg_ = 57, bandwidth = 1080 Hz/pixel, linear k-space encoding, parallel imaging acceleration factor = 2, 34 reference k-space lines obtained in a separate heartbeat.

Retrospective, short axis, multi-slice cine CMR was performed with a temporal resolution = 40 ms, flip angle = 70°, bandwidth = 940 Hz/pixel, spatial resolution = 1.1 × 1.1 mm^2^, slice thickness = 6 mm.

The animals received a 0.1 mmol/kg intravenous injection of gadolinium contrast for LGE imaging (MultiHance; Bracco Diagnostics, Inc; Princeton, NJ). Imaging was performed 10 minutes after injection of contrast agent using an inversion time (TI) scout sequence to determine the inversion time to null myocardial tissue signal. LGE CMR was obtained using a 3D multishot phase-sensitive inversion recovery (PSIR) bSSFP sequence at spatial resolution = 1.2 × 1.2 mm^2^, flip angle = 50°, TE = 1.6 ms, TR = 3.2 ms. slice thickness = 2 mm, and parallel imaging acceleration factor = 2 [17].

### Histology

After ex vivo CMR, the heart was flash-frozen using liquid nitrogen in a 4.5 L cryogenic container (Fisher Scientific, Waltham, MA). The heart was sectioned into uniformly thick short axis slices using a commercial-grade prosciutto slicer (330 M; Berkel) obtaining slices of uniform thickness of approximately 2 mm to be matched to CMR studies. Slices were submerged in a PBS solution with 0.1 M triphenyl tetrazolium chloride (TTC) and incubated at 50 ° C for 15 min [18]. Slices were removed from TTC solution and mounted on glass slides with aqueous mounting media (Aquatex; EMD Millipore). TTC stained viable tissue deep red, distinguishing scar tissue and viable myocardium. Slides were imaged at 800 dpi resolution using an optical scanner (Perfection V700; Epson). Tissue sections from remote and infarct regions were selected for further histological analysis and fixed in 10% neutral buffered formalin. Tissue samples were stained with Masson’s trichrome. Collagen was stained blue whereas keratin, muscle fibers, and cytoplasm were stained red and nuclei dark red.

### Image analysis

Cine CMR image series were used to calculate indexed LV mass (Mass), wall thickness (WT), end-diastolic volume (EDV), end-systolic volume (ESV), ejection fraction (EF) and cardiac output (CO). Epi- and endocardial contours were drawn manually at ED and ES (excluding papillary muscles) using standard techniques (Qmass 7.5, Medis, Leiden, The Netherlands) [19, 20].

For all images, scar size was assessed using full width at half maximum (FWHM) thresholding in a mid-LV slice with visible enhancement on LGE (QMass) [20]. FWHM thresholds were determined by drawing two regions-of-interest (ROIs): one in an area of non-enhancement and normal wall motion and a second around the hyperintense myocardium and used to define the maximum signal for FWHM threshold. ROIs were not adjusted to include hypointense regions of hemorrhage. Residual blood pool or pericardial regions of high brightness were manually removed from the ROI. Scar size was reported as the ratio (%) of the infarct ROI volumes and remote myocardium volume.

Scar transmurality was computed as the ratio of hyperintense (infarct) to non-enhanced myocardium (%) in a mid-ventricular short axis slice. The myocardium was divided into six circumferential wedges and transmurality was reported for anterolateral and posterolateral segments in which infarction was observed (two of six segments). Scar transmurality <5% in a segment was excluded as noise.

Motion correction was used to align T1ρ and T2 images using optical flow estimation of the image deformations [8]. T1 and T2 mapping was performed using a 3-parameter model

$$ S= A{e}^{\frac{- TSL}{T_{1\rho}}}+ B, $$

where A and B are additional free parameters, and $\mathrm{T}\mathrm{S}\mathrm{L}$ the contrast evolution time (spin lock time) (MatLab, Natick, MA). Infarct T1ρ, T2 and native T1 (T1 in the absence of gadolinium contrast agent) were calculated from the thesholded region-of-interest (ROI) and remote or baseline myocardium from a normal region (QMass).

Contrast-to-noise ratio (CNR) was calculated for T1ρ, T2 and native T1:

$$ C N R=\frac{\varDelta TX}{\sigma \left( T{X}_{rem}\right)}, $$

where *∆TX*=*TX*_*inf*_–*TX*_*rem*_ and *TX*_*inf*_ (*TX*_*rem*_) was the mean relaxation time in the infarcted (remote) tissue and σ(*TX*_*rem*_) was the standard deviation of the relaxation times observed in remote myocardium [21].

### Statistics

Descriptive statistics were reported as mean ± standard deviation (SD). Comparison of statistical means was performed using 2-way analysis of variance. Correlations were assessed with Pearson’s r. Bland-Altman testing was performed to test for mean bias and variation between imaging methods [22]. Pairwise infarct size changes of CMR measurements were assessed with 1-way analysis of variance. Histological infarct size changes were assessed with Student’s *t*-test.

## Results

### Changes in hemodynamics and wall thickness

All pigs (*s* = 10) survived the 90 min ischemia and reperfusion study to their terminal CMR at 1 or 4 weeks. At 4 weeks post-MI, there was a 13.4 ± 5.4% reduction in EF compared to baseline and associated with a 22.7 ± 8.0 mL increase in ESV and a 24.2 ± 14.1 mL increase in EDV. End diastolic wall thickness decreased by 4.0% after 4 weeks (*p* < 0.05) and ΔWT and ΔLVEDV between baseline and 4 weeks were correlated (r = 0.90, *p* < 0.05). Additional details are reported in Table 1.

**Table 1:** Hemodynamics at baseline and post-infarction, mean ± standard deviation

	Baseline (*n* = 5)	1 Week (*n* = 10)	4 Week (*s* = 5)
LVEDV, mL	70.3 ± 8.7	85.2 ± 13.7	94.5 ± 11.9*
LVESV, mL	29.5 ± 6.8	45.5 ± 12.9	52.2 ± 10.3**
LVEF, %	58.4 ± 5.7	48.5 ± 6.8	45.0 ± 5.9*
CO, mL/min	4.4 ± 0.6	4.1 ± 0.6	4.1 ± 0.6
LV mass, g	57.7 ± 8.1	66.6 ± 8.6	65.1 ± 9.9
WT^a^, mm	5.0 ± 0.4	5.1 ± 0.6	4.8 ± 0.5*

^a^WT reported for mid-LV slice with visible scar on LGE

^*^ *p* < 0.05; ^**^ *p* < 0.01 (paired *t*-test, 4 week different from 1 week)

### T1ρ, T2 and T1 mapping

**Fig. 2:**
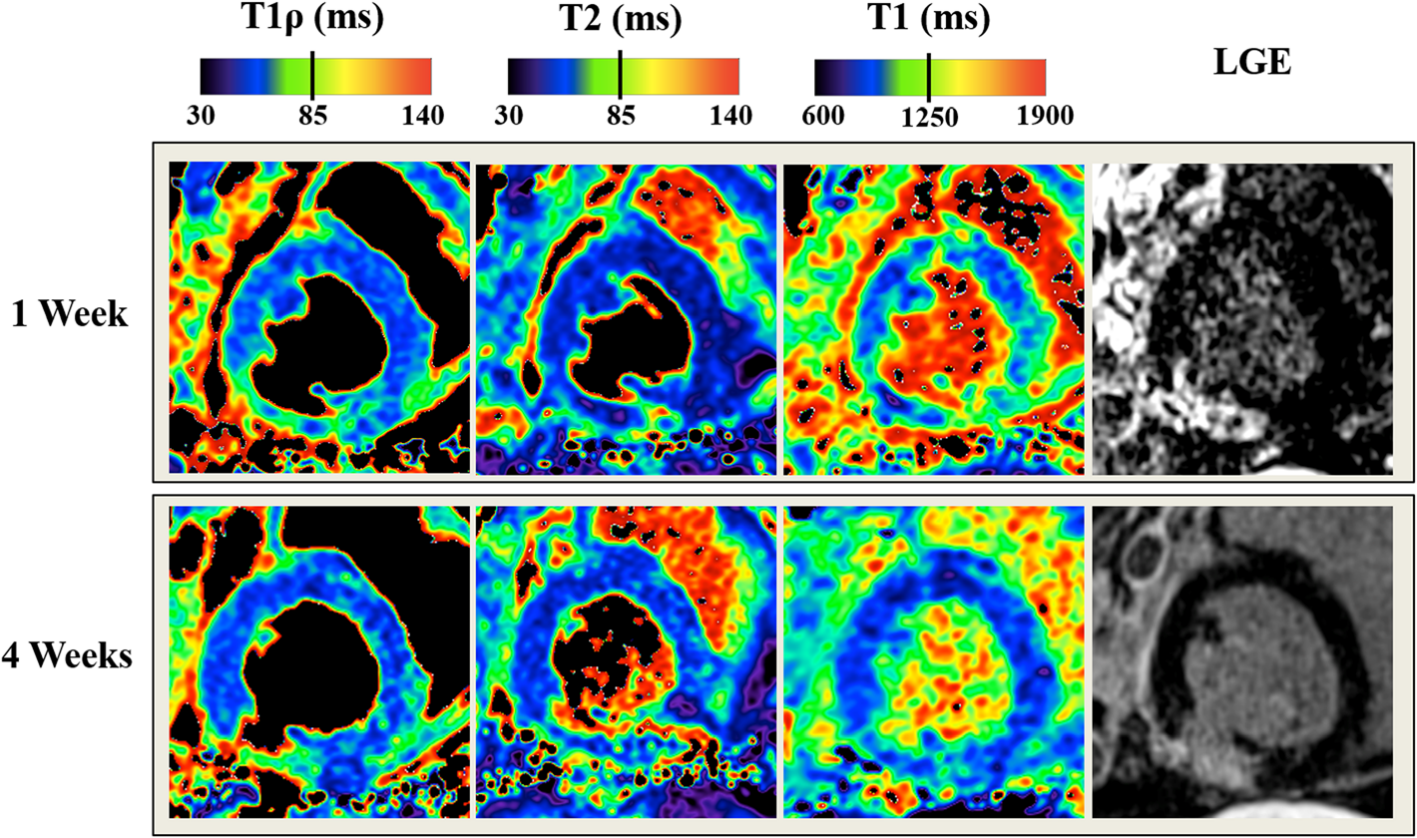
T1R, T2, T1 and LGE at 1 and 4 weeks in the same animal. The arrows in the LGE images indicate the hyperintense infarct region. Full LV coverage is shown in Additional file 1: Figure S1

Hyperintense regions were observed at the lateral wall on all T1ρ, T2, and T1 maps and in LGE images at 1 and 4 weeks, consistent with ischemic injury to the circumflex coronary circulation (Fig. 2; full LV coverage shown in Additional file 1: Figure S1).

**Fig. 3:**
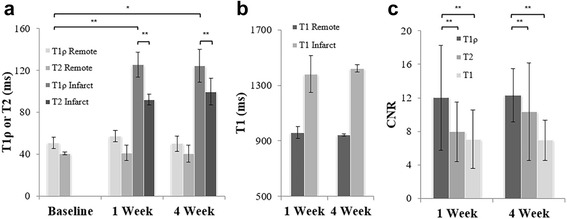
Relaxation times and contrast-to-noise ratio at 1 and 4 weeks after ischemia and reperfusion. **a** Relaxation times for T1ρ and T2 in healthy remote myocardium and infarcted myocardium and **b** Native T1 relaxation times in healthy remote myocardium and infarcted myocardium. **c** contrast-to-noise ratio of T1ρ, T2 and T1. * indicates *p* < 0.05 and ** indicates *s* < 0.01

As illustrated in Fig. 3, infarct T1ρ and T2 relaxation times were significantly increased at 1 and 4 weeks in infarcted myocardium in comparison to healthy myocardium prior to infarction with T1ρ = 50.9 ± 5.5 (baseline), 125.2 ± 12.1 (1 week) and 124.3 ± 15.6 ms (4 week) and T2 = 40.5 ± 7.3 (baseline), 92.1 ± 1.4 (1 week) and 99.6 ± 12.6 ms (4 week). T1 data was not acquired at baseline. T1ρ, T2, or T1 times were not significantly different from 1 to 4 weeks in the infarcted regions. In addition, there was no difference between remote myocardium at 1 or 4 weeks and healthy myocardium at baseline. ∆T1 (infarct-remote) = 421.3 ± 108.8 (1 week) and 480.0 ± 33.2 ms (4 week), ∆T1ρ = 68.1 ± 11.6 and 74.3 ± 14.2 ms, and ∆T2 = 51.0 ± 10.1 and 59.2 ± 11.4 ms. CNR_T1ρ_ = 12.0 ± 6.2 (1 week) and 12.3 ± 3.2 (4 week), CNR_T2_ = 8.0 ± 3.6 and 10.3 ± 5.8 and CNR_T1_ = 7.0 ± 3.5 and 6.9 ± 2.4.

### Infarct size

Infarct size was not significantly different for T1ρ, T1 and T2 compared to LGE (*ss* = 0.14) and, among animals who underwent both 1 and 4 week CMR, significantly decreased from 1 to 4 weeks (*s* < 0.01). Individual infarct size changes were ΔT1ρ = −3.8%, ΔT1 = −3.5% and ΔLGE = −2.8% from 1 to 4 weeks (Table 2). No change in infarct size was observed for T2.

**Table 2:** Infarct size and transmurality, mean ± standard deviation

	T1ρ	LGE	T2	T1	Ex vivo
1 Week					
Infarct Size, %	12.4 ± 2.7	11.8 ± 2.6	9.9 ± 2.8	14.0 ± 5.3	8.8 ± 2.3
Transmurality, %	50.3 ± 16.8	42.6 ± 19.9	31.1 ± 15.2	48.6 ± 26.6	N/A
4 Week					
Infarct Size, %	8.6 ± 2.3*	9.0 ± 2.9*	9.8 ± 2.5	10.5 ± 2.3*	10.1 ± 4.0
Transmurality^a^, %	32.8 ± 18.8	45.5 ± 17,8	47.8 ± 15.5	33.1 ± 22.6	N/A

^a^Transmurality reported for infero- and anterolateral myocardial segments (two segments of six total) for segments with transmurality > 5%

*p < 0.05 (paired *t*-test, 4 week different from 1 week). Transmurality not reported ex vivo.

**Fig. 4:**
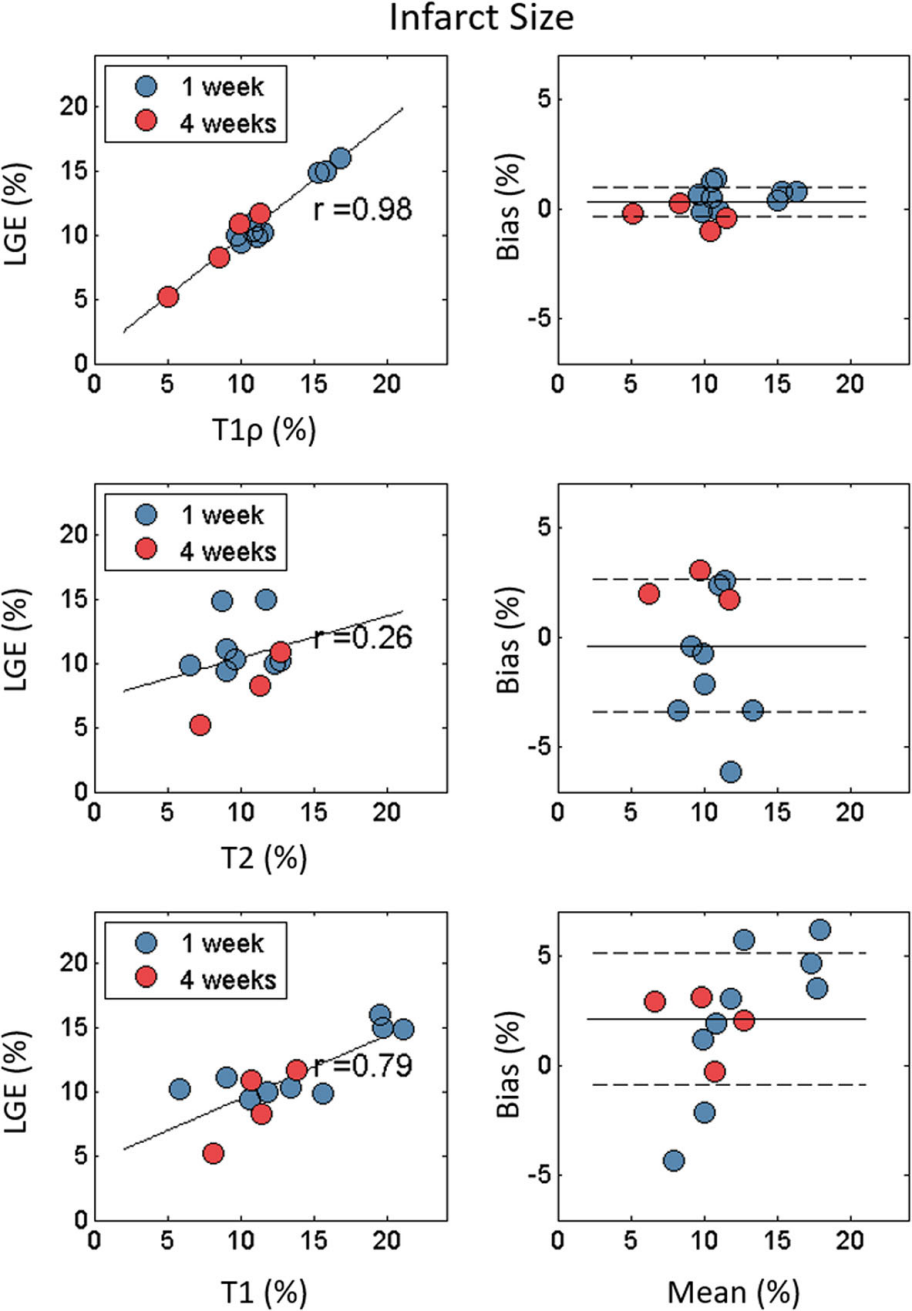
Infarct size correlation and Bland-Altman graphs for T1ρ, T2 and T1 in comparison to LGE. There was excellent correlation between T1ρ and LGE scar size, good correlation between T1 and LGE, and poor correlation between T2 and LGE.

There was excellent correlation of infarct size between T1ρ and LGE (r = 0.98, *p* < 0.001), good correlation for T1 and LGE (r = 0.79, p < 0.01) and poor correlation for T2 and LGE (r = 0.26, p = 0.44) (Fig. 4). Infarct size mean bias between T1ρ and LGE = 0.3, T2 and LGE = −0.4 and T1 and LGE = 2.3.

**Fig. 5:**
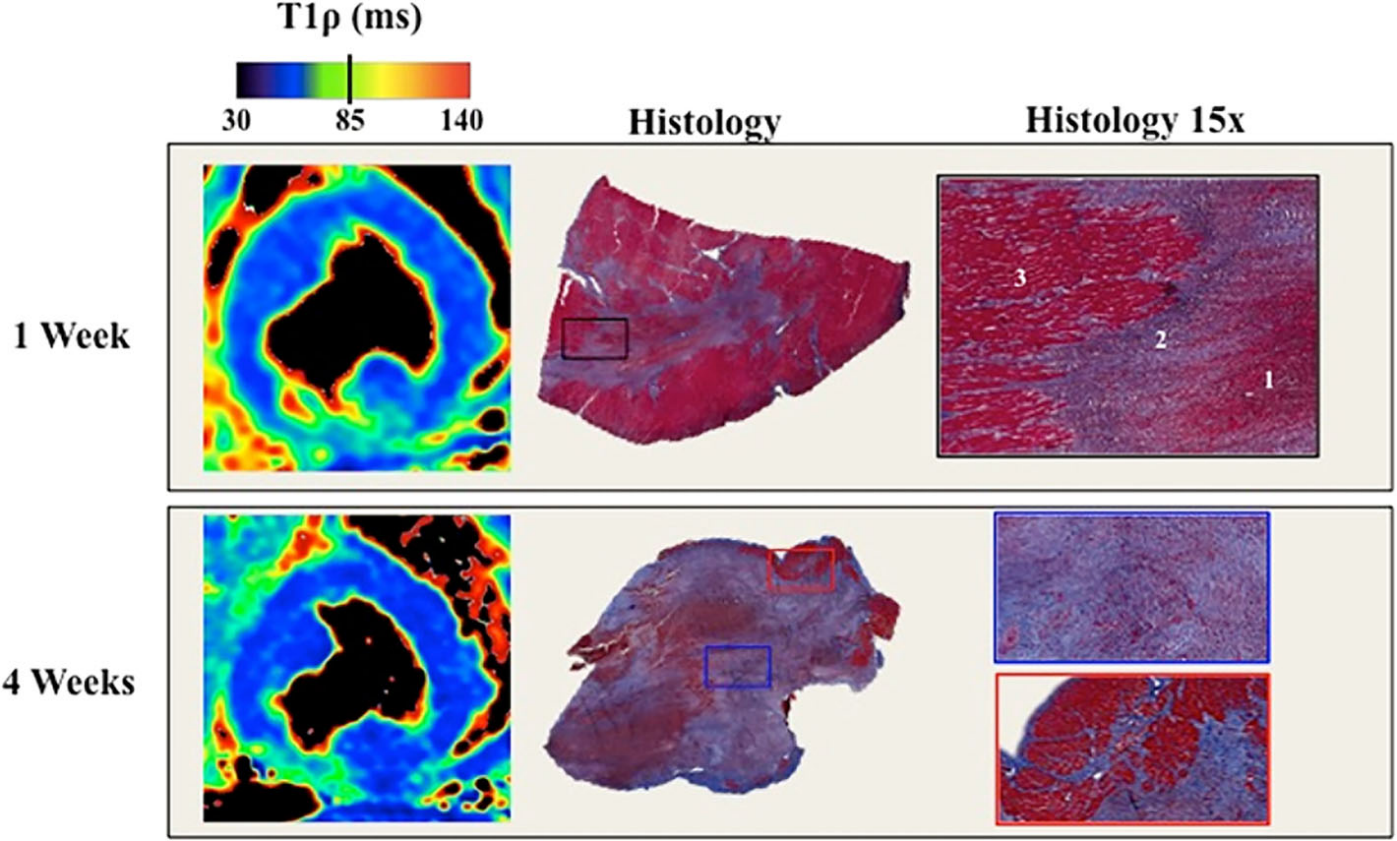
In vivo CMR and histology from two pigs at 1 and 4 weeks post-infarction. TTC staining shows an infarct with a hemorrhage core at the anteroseptal myocardium at 1 week post-MI. In the fibrotic regions, there was intermixed granulation tissue, dense fibrosis and myocardium and mature scar at 4 weeks. There was no difference in infarct size at 1 or 4 weeks compared to histology. *1. Hemorrhage core and intermixed granulation tissue; 2. Infarcted myocardium; dense fibrosis; 3. Remote myocardium.*

Infarct size at histology was 8.9 ± 2.3% at 1 week and 10.1 ± 4.1% at 4 weeks. There was no significant difference in infarct size at 1 or 4 weeks or as compared to any CMR measurement (p < 0.41; Fig. 5).

### Infarct transmurality

Infarct transmurality was not significantly different for T1ρ, T1 and T2 compared to LGE (*s* = 0.7) and was unchanged from 1 to 4 weeks (*p* = 0.15).

**Fig. 6:**
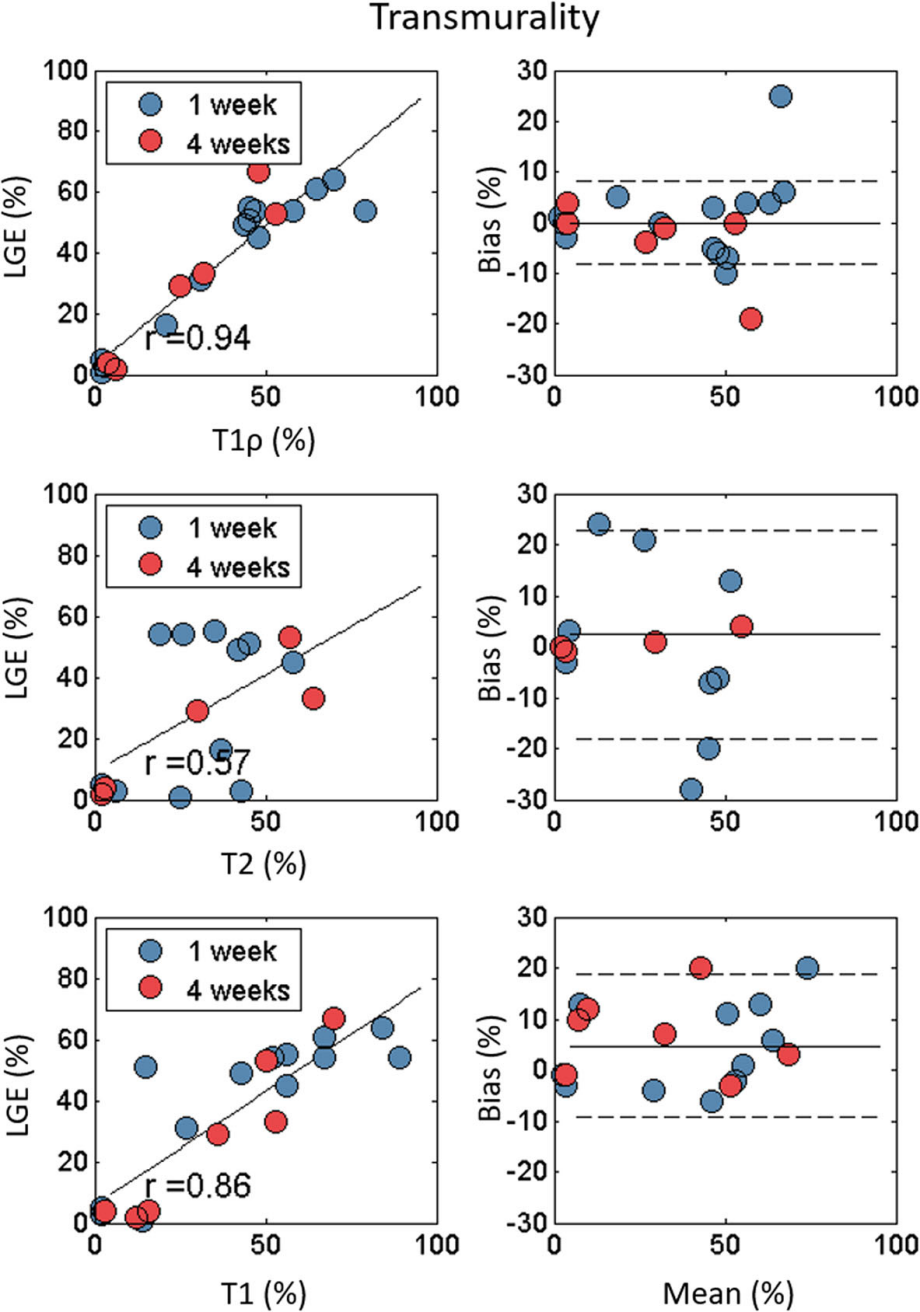
Infarct transmurality correlation and Bland-Altman graphs for T1ρ, T2 and T1 in comparison to LGE. There was excellent correlation between T1ρ and LGE scar transmurality.

There was good correlation of infarct transmurality between T1ρ and LGE (r = 0.94; *p* < 0.001), T1 and LGE (r = 0.86; *p* < 0.001) and moderate correspondence between T2 and LGE (r = 0.86; p = 0.02) (Fig. 6).

## Discussion

This study investigated for the first time endogenous contrast T1ρ, T1 and T2 mapping at 1 and 4 weeks after ischemia and reperfusion and their relationship with infarct size as determined by LGE and histology. The main findings were that (1) T1ρ values had higher relaxation time-dependent change than T2 and contrast-to-noise ratio compared to T1 and T2 in the infarcted myocardium; (2) there was a decrease in infarct size from 1 to 4 weeks on T1ρ, T1 and LGE CMR; and (3) T1ρ infarct size was better correlated with LGE than T1 or T2 and that T2 in particular was poorly correlated. Improved infarct contrast-to-noise ratio on T1ρ may explain the better correlation with LGE and histological infarct size and transmurality than T1 and T2.

### Endogenous contrast in infarcted myocardium

T1ρ is increased in acute and chronic myocardial infarction [6, 7, 11, 13], but little is known about the evolution of T1ρ post-infarction and its relationship with scar size. T1ρ progressively increased in non-reperfused MI in mice at 1, 3 and 7 days post-infarction, but remained constant from 7 – 20 days [13]. We did not examine the earliest times post-infarction (<24 h), however, we found significant T1ρ, T2 and native T1 changes at 1 week post-MI and confirmed histologically that these changes corresponded to early scar formation and extensive collagen deposition. This suggests that endogenous contrast changes at 1 week may be in part associated with formation of collagen, increased water mobility in collagen, and not necessarily increased myocardial water content in edema.

Furthermore, while T2 and native T1 are believed to be biomarkers for myocardial edema in the area-at-risk after acute MI [2, 23], recent studies have more closely examined this paradigm, suggesting that (at least) T2 has complex temporal behavior early post-infarction. Fernandez-Jimenez, et al. and Carrick, et al. reported two waves of edema with histological quantification of myocardial water content and associated it with bimodal T2 evolution at <24 h and at 1 week in pigs [24, 25]. Kim, et al. also reported in canines that T2 was not sensitive to the area-at-risk at 3–5 days week post-infaction [26]. While we did not directly measure area-at-risk using an approach such as injected microspheres, there was no evidence that any endogenous contrast was associated with at-risk myocardium at 1 week (was not larger than LGE infarct size). Furthermore, 90 min of ischemia-reperfusion in this animal model would generate significant myocardial salvage (between 5–15% salvage) [27], which was not observed by inspection of differences between endogenous contrast and LGE infarct size.

T2 had reduced correlation with LGE as compared to T1ρ and T1, which may be partly explained by differences in the way post-infarction hemorrhage affects endogenous contrast. Degradation of hemoglobin byproducts in the hemorrhage in T2 and T2* CMR studies in patients and animals contribute to increased magnetic susceptibility-induced dephasing on CMR [21, 28]. Accurate infarct size measurement should manually correct for hemorrhage, which is not labeled properly by semi-automatic thresholding. In the case of T1ρ CMR, the spin locking pulse may mitigate the effects of magnetic susceptibility-induced dephasing of the transverse CMR signal. Thus T1ρ may more accurately reflect total infarct size on semi-automated thresholding. Manual correction may introduce bias and inadvertently label remote or salvaged myocardium, so we did not manually correct semi-automated thresholding.

Another reason for the poor T2 agreement with LGE is that different methods of preparation may give different T2, e.g. preparations with adiabatic refocusing pulses instead of a non-selective Hahn spin echo [29]. The frequency and amplitude modulation of the adiabatic refocusing pulse, refocusing pulse spacing, and the peak amplitude of the adiabatic pulse may improve T2 results by partly suppressing hemorrhage-induced signal dephasing in a similar fashion to T1ρ [30, 31]. In addition, T1ρ is higher than T2 in almost all situations in which it has been measured in human tissues, including the myocardium [9, 11, 13]. This is attributed to the T1ρ dispersion, which is the variation in T1ρ with the preparatory pulse amplitude B_1_. As the amplitude B_1_ approaches zero, T1ρ approaches T2 (with the additional assumption that there is a refocusing pulse between the SL pulses). In our comparison of T1ρ, T2 and T1, we used identical readout sequences to eliminate a major cause of measurement variation. In addition, we matched the time between adjacent T2 or T1ρ preparations (4 heartbeats) and discarded the first scan because of variations in T1 recovery between preparations.

### Scar size and scar transmurality

Infarct size is expected to decrease from sub-acute to chronic post-infarction times [32]. T1ρ, T1 and LGE showed a decrease in infarct size from 1 –s 4 weeks, but a similar change was not observed histologically. This is likely explained by the small number of animals and the absence of histological data from the same animal at 1 and 4 weeks. The use of serial imaging of the same animal and measurement of infarct at the same mid-ventricular position in vivo, likely permitted this detection by in vivo mapping. Yorkshire swine have well-characterized coronary artery anatomy of a similar size and distribution to adult humans. While there is considerable variability in the size, orientation and vascular bed associated with the branches of the circumflex artery in pigs, the overall infarction size was prospectively determined by direct inspection of the circumflex and branch coronary artery distribution at the time of surgery. Therefore the pig infarction model produced reproducible infarct size and spatial distribution and localized to the inferior posterolateral wall.

We found that T1ρ and T1 infarct transmurality was correlated with LGE CMR at 1 and 4 week post-MI. However, differences in T1ρ infarct transmurality might be explained by the varying acquisition times in the cardiac cycle. Although infarct size is not influenced by cardiac cycle, scar transmurality (derived from LGE CMR) has shown to vary between end-diastolic and end-systolic assessment [33]. LGE MR images were derived during end-diastole, however T1ρ images were derived during end-systole, which is a plausible explanation for the differences in scar transmurality.

## Conclusion

T1ρ CMR is increased in myocardial infarction compared to T2 and has improved contrast-to-noise ratio compared to T1 and T2. Infarct size and transmurality on T1ρ and native T1 endogenous contrast was correlated with LGE CMR and thus may be useful as endogenous contrast CMR methods in ischemic patients who cannot receive contrast agents.

## Additional file

Additional file 1: Figure S1. Supplementary figure 1. (TIF 2430 kb)

## Abbreviations

CMR: Cardiovascular magnetic resonance
CNR: Contrast-to-noise ratio
CO: Cardiac output
EDV: End-diastolic volume
EF: Ejection fraction
ESV: End-systolic volume
FWHM: Full width half maximum
LGE: Late gadolinium enhancement
LV: Left ventricle
MI: Myocardial infarction
ROI: Region-of-interest
WT: Wall thickness
