# Erratum to: Assessment of myocardial injury after reperfused infarction by T1ρ cardiovascular magnetic resonance

**DOI:** 10.1186/s12968-017-0354-6

**Published:** 2017-03-27

**Authors:** Rutger H. Stoffers, Marie Madden, Mohammed Shahid, Francisco Contijoch, Joseph Solomon, James J. Pilla, Joseph H. Gorman, Robert C. Gorman, Walter R. T. Witschey

**Affiliations:** 1 Department of Radiology, Perelman School of Medicine, University of Pennsylvania, 1 Silverstein 3400 Spruce Street, Philadelphia, PA 19104 USA; 2 Gorman Cardiovascular Research Group, University of Pennsylvania, Philadelphia, PA USA

## Erratum

In the original version of this article [1], published on 15 February 2017, the author names were incorrectly displayed.

Originally the author names have been published as:

- Joseph H. Gorman
- Robert C. Gorman III

The correct author names are:

- Joseph H. Gorman III
- Robert C. Gorman

The original publication of this article has been corrected. The publisher apologizes to the readers for the inconvenience caused.

## References

[1] Stoffers (2017). Assessment of myocardial injury after reperfused infarction by T1ρ cardiovascular magnetic resonance. J Cardiovasc Magn Reson.

