# Supplementary figures and images for: Assessment of myocardial injury after reperfused infarction by T1ρ cardiovascular magnetic resonance
Source: J Cardiovasc Magn Reson. 2017 Feb 15;19:17. doi: 10.1186/s12968-017-0332-z (PMC5310026; doi:10.1186/s12968-017-0332-z)

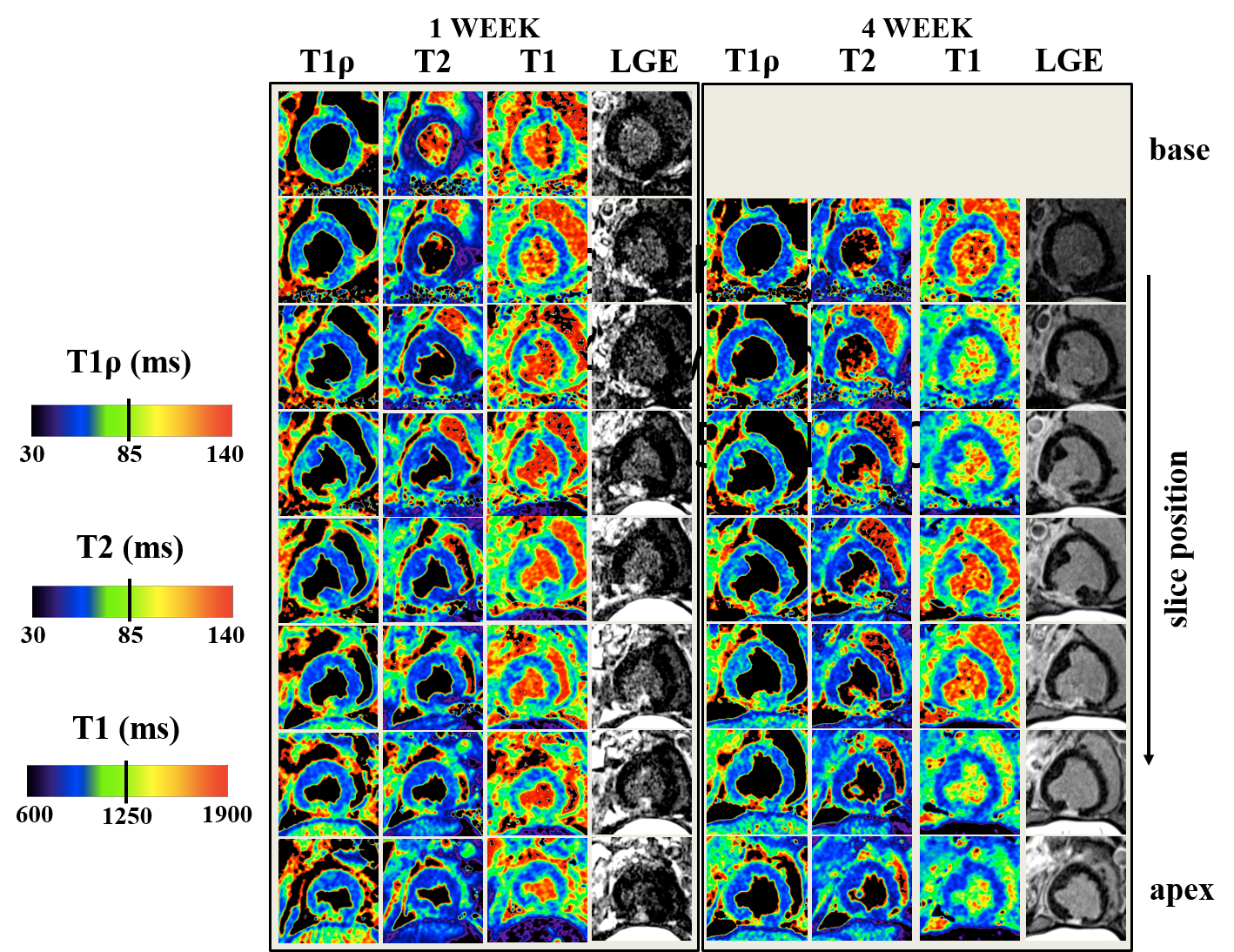

Supplement: Additional file 1: Figure S1. — Supplementary figure 1. (TIF 2430 kb) [file 12968_2017_332_MOESM1_ESM.tif]
